# Early Childhood Development policy in Chile: Progress and pitfalls supporting children with developmental disabilities toward school readiness

**DOI:** 10.3389/fpubh.2022.983513

**Published:** 2022-10-12

**Authors:** Cecilia Breinbauer, Verónica Vidal, Pamela Molina, Caterina Trabucco, Lina Gutierrez, Miguel Cordero

**Affiliations:** ^1^Department of Child and Adolescent Development, Center for Healthy Development, Seattle, WA, United States; ^2^Department of Epidemiology and Health Studies and School of Speech and Language Pathology, Universidad de los Andes, Santiago, Chile; ^3^World Federation of the Deaf, Helsinki, Finland; ^4^Community Organization Multiverso Phi, Santiago, Chile; ^5^Arica Autism Association, Arica, Chile; ^6^Centre for Research in Food Environments and the Prevention of Chronic Diseases Associated With Nutrition, Institute of Nutrition and Food Technology, University of Chile, Santiago, Chile

**Keywords:** developmental disabilities, children's mental health, policy, government investments, inclusive education, Chile, Autism, Early Childhood Development

## Introduction

There is now evidence indicating that first 5 years of life are of major importance for learning and health across life course (1, 2). This period is key in providing detection and timely supports for children with developmental disabilities (3, 4). Because of this, many investments have been done around the world (5). Specifically for this article, we illustrate the case of Chile. This paper provides an overview of some investments in public health, social protection, and education that Chile has done in supporting children with developmental disabilities during early childhood. The authors also provide their opinion about progress as well as barriers affecting school readiness for children with developmental disabilities during the last decade.

## Background and Early Childhood Development (ECD) programs currently funded by Chile for children with disabilities

In 2000, UNESCO considered that Chilean educational results were poor compared to countries with similar economic development and that the large gaps between rich and poor groups were alarming (6). However, the absence of national data on developmental delays in children made it impossible to identify the fraction of the population in need of preventive or supportive services. In 2005, the National Survey of Health and Quality of Life integrated a parent report on the developmental milestones of children from 3 months to 5 years. The objective was to estimate the magnitude of developmental delays so that the health, education, and social sectors could plan their budgets and programs (7). Since then, Chile has made significant investments in policies aimed at the early detection, prevention, and services for developmental disabilities in children under 5 years of age (8).

Since 2005, a new policy guarantying by law to the entire population, opportunity to access health services and financial protection—through Explicit Guaranties from a list of health conditions, as well as extra support for high-cost eligible medical treatments, increased access to medical treatments that might support some children with disabilities (9, 10). For example, any child born before 32 weeks of gestation or born with <1,500 gr has guaranteed access to hearing screening. If the screening reveals significant hearing deficit (more than 35 decibels), children are eligible to receive headphones, cochlear implants, and speech and language therapy. Since 2013, children under 4 years old with moderate, severe, or profound deafness (more than 40 decibels hearing deficit) have also access to headphones, cochlear implants, and therapy (11).

Another action taken in the health sector is the integrated health guideline for primary health care providers, recommended by the Ministry of Health (12). It includes a chapter for children with special needs, with special attention to Down Syndrome and Autism. This includes 24 services guaranteed for children with Down Syndrome during their first 5 years of life. The section for autism has a guideline for autism screening between 16 and 30 months of age, using the M-CHAT-R/F, validated for Chile, with high sensitivity and specificity (13). Chile started collecting M-CHAT-R/F data from all public health services in 2019. Unfortunately, COVID-19 restrictions reduced significantly screening services since 2020, making it hard to assess its impact.

Regarding education, Chile has been gradually implementing inclusive education, starting at the pre-kinder level since 2009 (14). Public schools receive financial support to include children with developmental disabilities (known as “permanent special needs”) in regular schools. The Ministry of Education provides financial supplement for every child with developmental disabilities integrated into regular education, with a cap of 2 students per class. Schools who integrate deaf or blind children in small class sizes (maximum 8 children) receive an additional financial supplement (15). Access to inclusive education for children with developmental disabilities under 5 years old has increased (16).

In 2007, a cross-sectoral system of integrated services through the Social Protection sector was implemented. The national Early Childhood Development policy “Chile Crece Contigo” (ChCC, Chile grows with you) coordinates activities offered across nine ministries, from the prenatal period up to 9 years old (17, 18). In 2019, ChCC identified lack of timely services for eligible children with developmental disabilities (from 60% most socially vulnerable households), including autism, and developed pilot programs for supporting children with developmental disabilities called now Inclusive Rooms in 21 communities (“comunas”) across the country (19). This program finances the training and services of interdisciplinary teams, including speech and language pathologists, occupational, and/or physical therapists, who educate the parents and provide direct developmental services for children under 4 years of age. This pilot program also coordinates benefits that children with the national disability credential (issued by the National Service of Disability, SENADIS) can access. Example of benefits that can support children's readiness for school include assistive communication devices, such as tablets with speech generating devices for non-speaking autistic children over 4 years old, Braille typewriters, as well as other assistive technology (20).

## Discussion: Pitfalls and suggestions

Despite the large progress implementing ECD policies, Chile has currently not reported a national indicator on proportion of children under 5 years of age who are developmentally on track in health, learning, and psychosocial well-being. The governmental related website indicates that data is being developed, studied, or analyzed since 2019 (21) but indicator still is not available. Moreover, lack of randomized evaluations of several interventions scaled up in Chile makes difficult to recognize the cost-effectiveness of this large investments on child outcomes.

Following on a comparison of two health surveys separated by 10 years, Chilean government reported a massive reduction from 25% in 2006 to 11% in 2017 on developmental delay rates after implementing ChCC. However, only children at age 3 years old group exhibit these differences and no significant changes are observed at other age groups (22). On the other hand, experimental evidence from a large, randomized trial on a parenting intervention showed robust effects on reducing language developmental delays and lower rates of socioemotional developmental delays on families who were offered parenting classes in primary health care using the Canadian well-known Nobody's Perfect Parenting Program that was adapted to the Chilean culture (23). However, take up of the program was still small and generalizability to children with developmental delays was not possible because they were listed among the exclusion criteria of the target population.

Furthermore, we could not find a rigorous study following up children from lower socioeconomic background with cochlear implants and their school readiness, placement, and educational outcomes. We know that access to language for deaf people with developmental disabilities in a linguistically accessible environment, adapted to their communicative needs—including the use of their national sign language, is both a basic need and a fundamental human right (24). This aspect should be strengthened in the available programs.

Lissi et al. (25) included among their recommendations to the Ministry of Health, that early detected deaf children and their parents would be supported also by trained deaf psychologist and deaf educator during the early years, ensuring access to Chilean sign language and culture. The absence of this early support contributes to language deprivation with a profound impact on the quality of their educational inclusion and overall future development (24, 25). Currently, eligible deaf children under 4 years old are being supported by the new Inclusive Rooms, coordinated by ChCC. We encourage ChCC representatives of the Inclusive rooms to take Lissi et al.'s recommendations and to develop plans that exist in place to conduct a rigorous study to evaluate the impact of the pilot programs for children with developmental disabilities. Such plan should assess not only children's school readiness but also the readiness of schools to receive and support the children transitioning from this program to formal education (26).

Another barrier is the fragmentation of data collection. Chile's system to obtain a disability credential, through SENADIS, does not seem to have a publicly available database with the number of unidentified children who obtained the disability credential, disaggregated by diagnosis, age, gender, socio-economic status, and benefits provided, that can be analyzed for planning. In other words, no data, no problem, no action. Moreover, a recent study evaluating access of children with disabilities to services, identified that families are reluctant to obtain the national disability credential for their children. They described the process as cumbersome, slow and feel afraid of stigmatization (27). In addition, the information regarding benefits for children under 5 years of age in the SENADIS website is difficult to understand because is not presented in a friendly and accessible way.

In addition to the impact evaluation, and fragmentation, another pitfall of Chilean investments for school readiness of children with developmental disabilities is the lack of involvement of the strong disability community in Chile. It will be highly desirable that the Chilean disability community could help shaping the design of services, including an evaluation component, for young children with disabilities. Chile has a strong deaf community and a growing autism community. Current best practices recommend Community-Based Participatory Research involving scientific professionals and experts by experience working together in developing, implementing, and disseminating research (28). “Nothing about us without us”^1^ has become an expression that communicates that “no decision that influences people with disabilities should be made without their participation.” This movement provided the ethical basis, within international human rights, in calling State members to guarantee the participation of people with disabilities in all aspects of public policy (29). Neurodivergent communities point out the need to generate spaces for literacy, awareness, and development of knowledge about neurodivergences through instances of dialogue and co-creation of projects that include representative actors of existing neurodivergencies (30).

Lastly, the current new government administration has expressed interest in increasing funding for mental health. We hope that this very much needed attention to this important area, “no health without mental health” (31), includes also young children with disabilities. There is evidence that states that deaf persons show higher levels of anxiety and depression compared to the general population (32, 33). Also, the recent Lancet commission on Autism identified that anxiety among autistic children starts in infancy (Figure 2, page 275) (34). Pukki et al. (35) replied to the Lancet commission on autism, sharing the autistic perspectives on the future of clinical autism research. Among their recommendations, the autistic authors urge to focus more resources on mental health support, among other (35, 36). It is not clear to us how Chile's ECD program will support the transition of children with disabilities to schools, where bullying starts early and children with disabilities are often targets, affecting their learning and well-being. Currently, there is new evidence that neurodivergent children are at more risk of co-associated anxiety, depression, and suicide. School exclusion and bullying can be a modifiable factor (37). In addition, there are reports of high stress levels within the family environments of children with disabilities (38). It is a priority to include mental health support and services for parents and caregivers within national early childhood programs.

To have more inclusive communities, and therefore, better mental health of children with neurodevelopmental disabilities, it is important to focus on acceptance, significantly changing current practices, shifting from deficits to strengths-based approaches. We propose that a good way to start is to work on awareness and changes of pathologist and ableist^2^ views of disabilities (39). Chile has done significant progress in Early Childhood Development policies and programs. Children with disabilities are being included with more targeted services in the last few years. However, we still have a very ableist view of approach. Their lives are worthy as they are, and this needs to be highlighted. It is necessary to address disability as part of human diversity, where the design of health and education programs incorporate dignified and respectful perspectives of their identity, from those who live the experience. We hope that our views shared in this opinion article help to strengthen Chile's programs and be a model for the world also in this area.

## Author contributions

All authors listed have made a substantial, direct, and intellectual contribution to the work and approved it for publication.

## Conflict of interest

The authors declare that the research was conducted in the absence of any commercial or financial relationships that could be construed as a potential conflict of interest.

## Publisher's note

All claims expressed in this article are solely those of the authors and do not necessarily represent those of their affiliated organizations, or those of the publisher, the editors and the reviewers. Any product that may be evaluated in this article, or claim that may be made by its manufacturer, is not guaranteed or endorsed by the publisher.

## Footnotes

^1^“Nothing about us without us” is a phrase used by South African disability rights activists Michael Masutha and William Rowland in the 1990s; and then in 1998, J. Charlton begins to use and spread it.

^2^According to Nario-Redmond, ableism is the discrimination of and social prejudice against people with disabilities based on the belief that typical abilities are superior (39).
